# Characterization of a Resistively Multiplexed SiPM Readout for Position-Sensitive Scintillating Fibre Radiation Detectors

**DOI:** 10.3390/s26206531

**Published:** 2026-10-15

**Authors:** Klemen Žagar, Gregor Košir, Matjaž Vencelj, Eva Lovšin, Tobe Mulder, Jürgen Gerl, Hao Huang, Guang-Shun Li, Jelena Vesić

**Affiliations:** 1 Jožef Stefan Institute, Jamova cesta 39, 1000 Ljubljana, Slovenia; 2 Jožef Stefan International Postgraduate School, Jamova cesta 39, 1000 Ljubljana, Slovenia; 3 Faculty of Mathematics and Physics, University of Ljubljana, 1000 Ljubljana, Slovenia; 4 FAIR GmbH, Planckstraße 1, 64291 Darmstadt, Germany; 5 Institute of Modern Physics, Chinese Academy of Sciences, 509 Nanchang Rd., Lanzhou 730000, China

**Keywords:** silicon photomultiplier, SiPM, scintillating fibre detector, radiation sensor, resistive multiplexing, charge division, position reconstruction, active stopper, FIMP, DESPEC, GSI/FAIR

## Abstract

Active stoppers based on scintillating fibres provide fast signal formation, fine segmentation and compact geometry. On the other hand, their implementation requires a readout that can handle many silicon photomultiplier channels and a wide range of light intensities. We present a laboratory characterization of a resistively multiplexed SiPM readout for the Fibre IMPlanter concept. The readout combines 16 SiPM input nodes into four analog outputs. Localized pulsed light excitation was used to scan individual SiPM positions, and the four signals were processed by baseline subtraction, charge integration and normalized charge division reconstruction. In an intermediate signal range, the reconstructed position plane shows distinguishable clusters for the individual inputs, indicating that spatial information is retained after a factor-of-four reduction in readout channels. At low amplitudes, the reconstruction is limited by baseline noise and reduced signal-to-noise ratio. At high amplitudes, the energy proxy signal response is affected by SiPM non-linearity and finite microcell occupancy, while position reconstruction accuracy is limited by distortions of the charge sharing. The results identify the signal range in which resistive multiplexing remains a practical compact readout option for SiPM-based scintillating fibre radiation detectors.

## 1. Introduction

Decay spectroscopy of exotic nuclei relies on event-by-event correlations between implanted ions and their subsequent radioactive decays. At GSI Helmholtzzentrum für Schwerionenforschung and the Facility for Antiproton and Ion Research (GSI/FAIR), the DEcay SPECtroscopy (DESPEC) programme was developed for nuclear-structure studies of exotic nuclei produced at the Fragment Separator (FRS) and, in future experiments, at the Superconducting Fragment Separator (Super-FRS) [1]. Together with the High-resolution In-flight SPECtroscopy (HISPEC) programme, DESPEC will exploit rare-isotope beams from the Super-FRS for in-beam and decay-spectroscopy studies at FAIR [2]. In a DESPEC experiment, the active stopper must determine the implantation time and position, detect subsequent β, α, isomeric or delayed particle decays, and provide sufficient energy information to separate different event classes. It is typically surrounded by high-resolution γ-ray, neutron, and fast-timing arrays. The present DESPEC workhorse, the Advanced Implantation Detector Array (AIDA), is based on double-sided silicon strip detectors (DSSSDs) and provides fine segmentation and a large dynamic range, but the decay event timing in the relevant operating mode is limited to a microsecond scale [3]. Fast timing performance is essential for the study of short-lived nuclei, high implantation rates, and decay chains in which random correlations can dominate the measured spectra. The need for segmentation follows directly from the implantation–decay correlation problem. If the active stopper were treated as a single sensitive volume, all decays detected within a chosen correlation time window would be possible candidates for previous implantations in that volume. By dividing the sensitive volume into many strips, fibres, pixels or voxels, the local implantation rate per detector element is reduced. In a simplified picture

(1) $$R_{\mathrm{segment}}≃\frac{R_{\mathrm{total}}}{N},$$

where $R_{\mathrm{segment}}$ is the mean implantation rate in one detector segment, $R_{\mathrm{total}}$ is the total implantation rate over the full sensitive detector volume, and *N* is the number of equivalent detector segments. For small random-correlation probabilities, the probability of an unrelated event within a time window Δt scales approximately as

(2) $$P_{\mathrm{random}}\propto R_{\mathrm{segment}}\Delta t.$$

Finer segmentation, therefore, improves the reliability of implantation decay correlations, but it also increases the number of readout channels. This trade-off is one of the main motivations for studying compact multiplexed readout schemes.

Scintillator-based active stoppers provide a complementary approach to silicon implantation detectors. Plastic scintillators offer fast signal formation and robust signal processing, and scintillating fibres extend this concept towards finer spatial granularity. Scintillating fibre detectors with silicon photomultiplier (SiPM) readout have been developed for compact tracking and timing applications [4,5], while single-photon avalanche diode (SPAD) array readout has also been demonstrated useful for scintillating fibre tracking and active-target applications [6]. The Fibre IMPlanter (FIMP) is being developed as a scintillating fibre active stopper in which orthogonal fibre layers are read out by SiPMs [7]. Plastic scintillating fibres are an established technology for fast, position-sensitive radiation detection. Due to their small cross section, they can be segmented on a sub-millimetre scale, and the fibre geometry permits detector structures with low material budget and geometric flexibility [4,8,9]. Representative implementations include the D0 and MICE scintillating fibre trackers, which used visible-light photon counters (VLPCs), and the LHCb Scintillating Fibre Tracker, which is read out by silicon photomultiplier arrays [4,10]. The advent of silicon photomultipliers has enabled the construction of compact fibre-detector modules that can be operated in magnetic fields, without the cryogenic requirements of VLPC-based systems [4,9]. In this concept, the fibre geometry provides the spatial segmentation, while the SiPM readout provides compact photosensor coupling and fast timing. The main readout challenge is that a detector with millimeter granularity requires a large number of SiPM channels.

The channel count is therefore a central sensor-design parameter. Large SiPM arrays increase the number of cables, front-end channels, digitizer inputs, bias connections, and data streams. Analog multiplexing reduces this complexity by combining several SiPM signals into a smaller number of outputs. Such readout reduction is widely used in position-sensitive radiation detectors and has been reviewed in the context of SiPM readout for nuclear imaging and positron-emission tomography (PET) systems [11,12]. Resistive charge division is one of the simplest implementations: the position is encoded in the relative charge collected at a small number of outputs [13,14]. Position sensitivity can also be implemented in the photosensor itself, either by connecting individual microcells to different output terminals or by an integrated resistive network read out at the chip corners [15,16,17,18,19]. The same principle has also been implemented with discrete position-weighted resistors on the sensor board, where a linear tile of ten SiPMs read out by two channels identifies the hit bar in a scintillator array [20].

Other representative SiPM readout and multiplexing approaches are summarized in Table 1. The principal trade-offs are between the degree of channel-count reduction and the amount of timing, amplitude, and position information retained by the readout.

For an active implantation detector, the readout must remain useful over a broad dynamic range. Implanted ions produce large light signals along their tracks and near the Bragg peak. Alpha particles produce relatively large local deposits and are expected to be distinguishable from beta particles by their larger local energy deposition. On the other hand, beta particles and secondary electrons generate smaller and more variable signals. Beta particles in the relevant DESPEC experiments have typical energies of the order of 1 MeV, while the deposited energy in one 0.5 mm fibre layer can be close to the detection threshold. The requirement that a beta particle reaches two adjacent orthogonal layers, therefore, sets a low-energy detection limit of the order of a few hundred keV in the present concept [7]. The usable range of the reconstructed response is determined by both the SiPM response and the multiplexing network. At low light levels, the reconstruction is limited by photon statistics, dark counts and electronic noise. At high light levels, the finite number of SiPM microcells introduces non-linearity and saturation-like behaviour; large signal currents and voltage drops in the resistive network may also modify the relative charge sharing [21,22]. The relevant question is therefore not only whether the multiplexing works but also over which signal range it remains a reliable position sensor.

In this work, we characterize a 16-to-4 resistively multiplexed SiPM readout for the FIMP detector concept. Resistive charge division multiplexing itself is an established technique. Early resistive position-encoding methods can be traced back to the Anger scintillation camera [23]. Later, Siegel et al. reduced the 64 output channels of a multi-channel PMT to four readout channels using three different resistor-network topologies [24]. Representative subsequent resistive charge division readouts were developed primarily for scintillator imaging and PET applications [14,24,25]. The present study instead focuses on the amplitude-dependent position response over the broad signal range relevant to an active implantation detector. The reconstruction performance is quantified using 99% probability content ellipses, nearest-neighbour separation, and amplitude-dependent centroid stability. Additional measurements address charge sharing, noise sensitivity, temperature dependence, and the practical operating window of the multiplexed readout. Localized pulsed light excitation is used as a controlled input to the SiPM array. This does not replace source or beam measurements with scintillating fibres, but it isolates the behaviour of the multiplexing network and reconstruction algorithm. The results identify the conditions under which a factor-of-four reduction in readout channels can retain position sensitivity in the tested laboratory configuration and provide a sensor-level evaluation of resistive multiplexing for compact SiPM-based radiation detectors.

**Table 1:** Comparison of representative SiPM readout and multiplexing approaches. Output-channel counts are representative of a 4×4 SiPM array and may vary with the specific implementation.

Readout Method	Typical Outputs	Encoding Principle	Principal Trade-Offs
Individual readout [12]	16	Each SiPM is acquired independently.	Retains the full channel information and avoids multiplexing ambiguity but requires the largest number of front-end and digitizer channels.
Row–column summing [12]	8	Signals from SiPMs are summed separately along detector rows and columns.	Provides moderate channel reduction with separate information from the two detector coordinates, but simultaneous events can introduce ambiguities.
Resistive charge division [13,14,25,26]	4	A resistor network partitions the SiPM signal among several outputs; position is inferred from their relative charges.	Provides strong channel reduction using comparatively simple passive circuitry, but RC loading, noise, component values and calibration can affect timing and reconstructed position.
Capacitive charge division [12,14]	4	Signal components are distributed among the position outputs through capacitive coupling.	Reduces resistive loading and can improve fast-signal response, but pulse undershoot, baseline recovery and capacitor matching must be considered.
Hybrid resistive–capacitive division [12,27]	4	Resistive and capacitive signal paths are combined for position encoding.	Provides a compromise between charge-sharing behaviour and fast signal response at the cost of increased circuit complexity.
Delay/time multiplexing [12]	2–4	Detector identity is encoded by controlled signal delays and recovered from signal arrival times or time differences.	Can achieve substantial channel reduction without relying on analog charge ratios but requires accurate timing extraction and delay calibration.

## 2. Materials and Methods

### 2.1. Scintillating Fibre Detector

**Figure 1:**
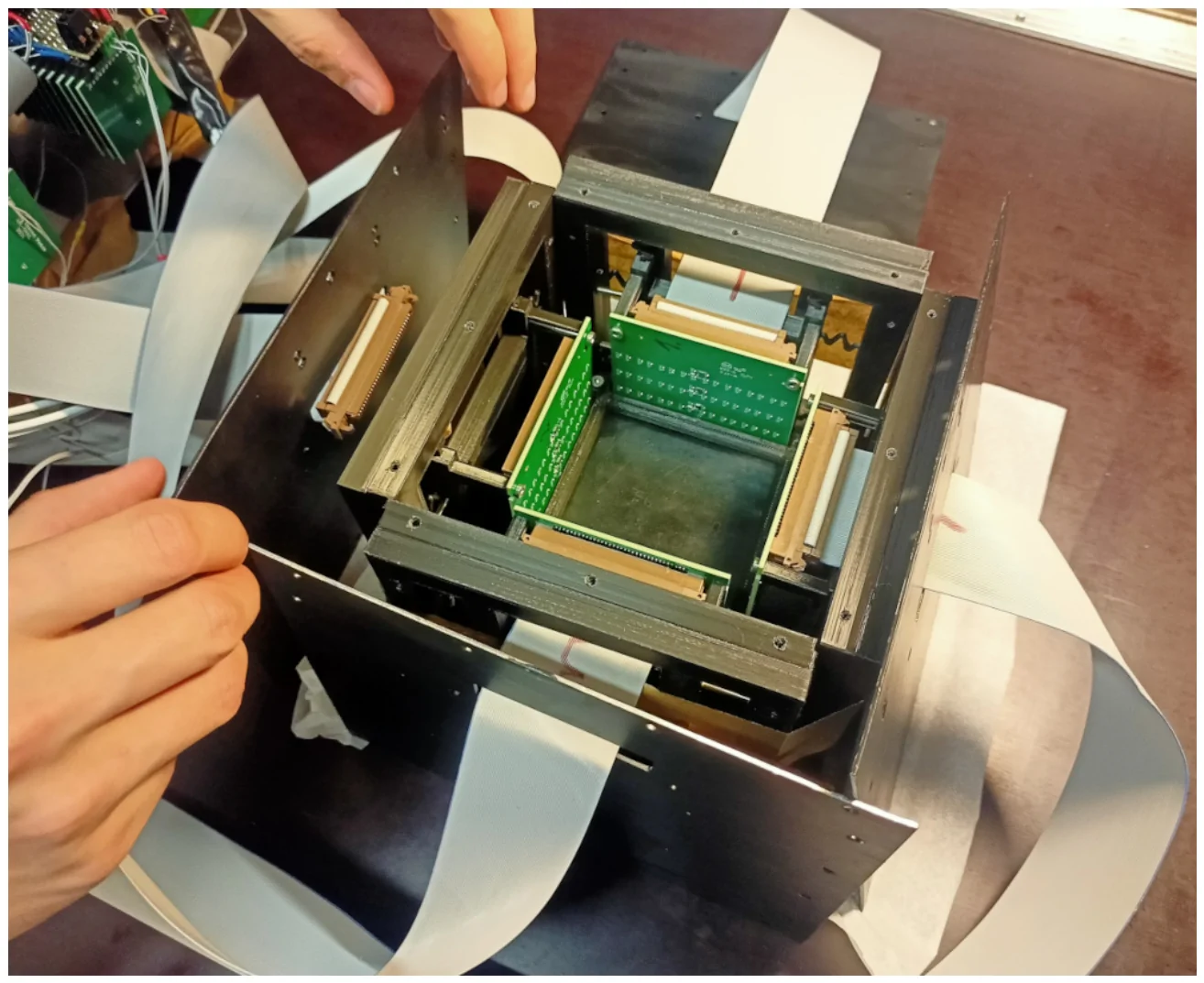
Photograph of the FIMP prototype. The active stopper concept uses orthogonal layers of scintillating fibres read out by SiPMs to provide fast timing and millimetre-scale position sensitivity for implantation–decay correlation measurements.

**Figure 2:**
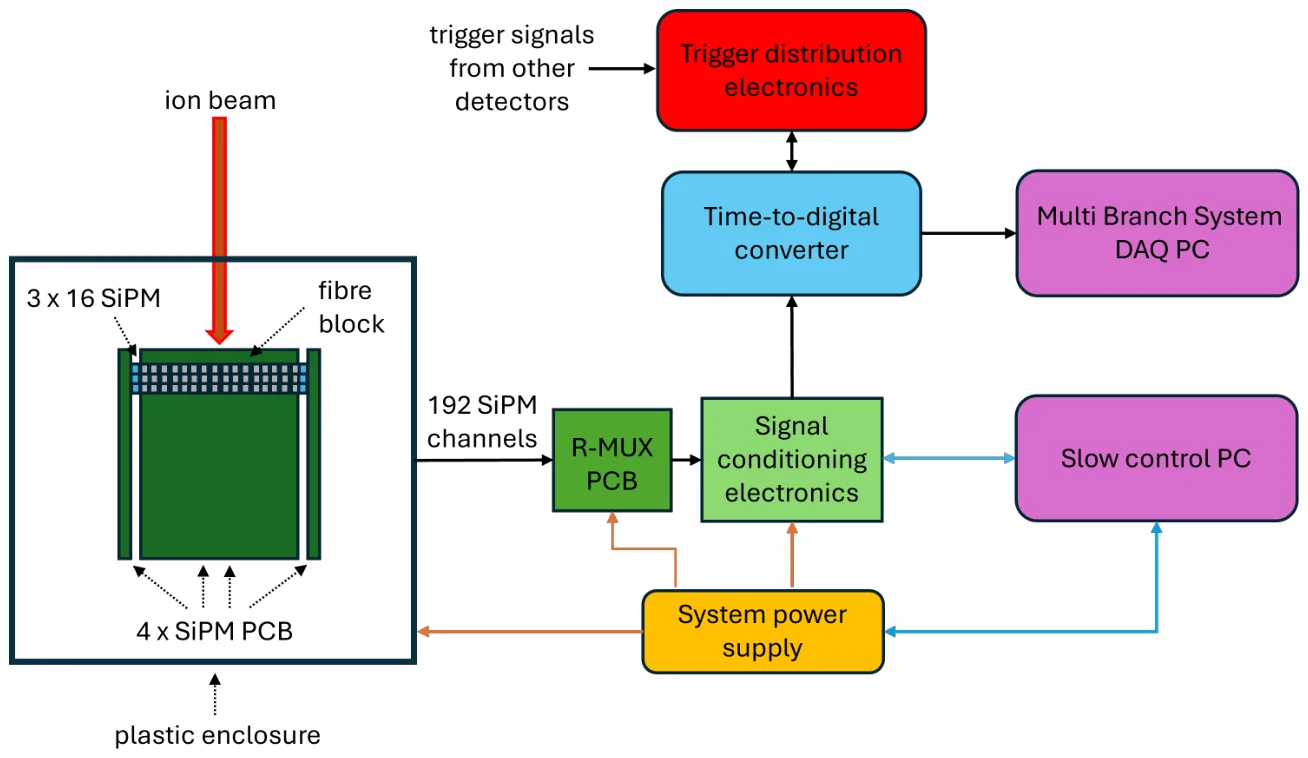
FIMP detector system schematic.

The FIMP detector is based on multiple layers of scintillating fibres arranged in orthogonal directions. The fibres act both as the active detection medium and as light guides that transport scintillation photons to SiPM photosensors. A photograph of the prototype is shown in Figure 1. An overall detector system schematic is shown in Figure 2. The orthogonal fibre geometry provides two independent projections of the event topology. When a particle deposits sufficient energy in each of two consecutive orthogonal layers, two-dimensional position information can be reconstructed.

The fibre pitch, approximately 0.5 mm in the present concept, is a compromise between spatial granularity and light output per channel. Reducing the fibre size improves granularity, but it also reduces the number of detected photons and makes the timing and threshold performance more sensitive to light yield, attenuation, optical coupling and SiPM photon detection efficiency. The same compromise is encountered in other scintillating fibre timing detectors [9,28].

The readout must therefore retain position information for weak beta-like signals while remaining usable for much larger implantation or alpha-like signals.

### 2.2. SiPM Response Relevant to Multiplexed Readout

An SiPM consists of many Geiger-mode microcells connected in parallel. Each fired microcell releases an approximately fixed charge determined by the effective cell capacitance and the overvoltage. The gain of one microcell can be written as

(3) $$G=\frac{C_{\mathrm{cell}}\Delta V}{q},\;\Delta V=V_{\mathrm{bias}}-V_{\mathrm{br}},$$

where $C_{\mathrm{cell}}$ is the effective microcell capacitance, *q* is the elementary charge, $V_{\mathrm{bias}}$ is the applied bias voltage and $V_{\mathrm{br}}$ is the breakdown voltage [21,22]. At low light levels, when the probability that more than one photon is detected by the same microcell during its recovery time is small, the total collected charge is approximately proportional to the number of fired microcells. This proportionality is the basis for using integrated SiPM signals as charge-like observables in a position reconstruction algorithm.

Several SiPM parameters enter directly into the multiplexed response. The photon detection efficiency (PDE) depends on the overvoltage and wavelength and can be written, in a simplified form, as

(4) PDE(ΔV,λ)=Fη(λ)ϵ(ΔV),

where *F* is the geometrical fill factor, η(λ) is the quantum efficiency and ϵ(ΔV) is the avalanche-triggering probability. The PDE determines the number of fired microcells for a given light input and therefore affects both the signal amplitude and the position resolution.

The response becomes non-linear when the number of photons arriving within the relevant recovery time is no longer small compared to the number of available microcells. In a simple saturation model for a short light pulse, the mean number of fired microcells is

(5) $$\langle N_{\mathrm{fired}}\rangle ≃M\left[1-\exp\left(-\frac{\mathrm{PDE}\left(\Delta V,\lambda \right)N_{\mathrm{ph}}}{M}\right)\right],$$

where *M* is the total number of microcells and $N_{\mathrm{ph}}$ is the number of incident photons. The former equation neglects cross-talk, afterpulsing and microcell recovery during the light pulse, but it captures the first-order loss of linearity caused by finite microcell occupancy. Equation (5) shows why the same readout can behave approximately linearly for moderate light pulses but distort at high amplitudes.

In a resistively multiplexed readout, SiPM non-linearity is especially important because the reconstructed coordinates depend on ratios of the four output charges. If one part of the readout network enters a non-linear regime earlier than another, the relative charge sharing changes, and the reconstructed position can shift. Noise mechanisms also affect the low-amplitude limit. Dark counts, afterpulsing and optical cross-talk add charge fluctuations that are not related to the scintillation or LED signal. These fluctuations affect both the total signal *Z* and the charge ratios used for position reconstruction.

Temperature is also a relevant control parameter. At fixed bias voltage, a change in temperature changes the breakdown voltage of an SiPM and therefore the overvoltage, which in turn changes the gain. The dark count rate also depends strongly on temperature [21,22]. A stable multiplexed detector, therefore, requires either temperature stabilization or a calibration that corrects the gain of the four outputs as a function of temperature. These considerations motivated the tests of signal amplitude, noise and temperature dependence described below.

### 2.3. Resistive Charge Division Readout Network

**Figure 3:**
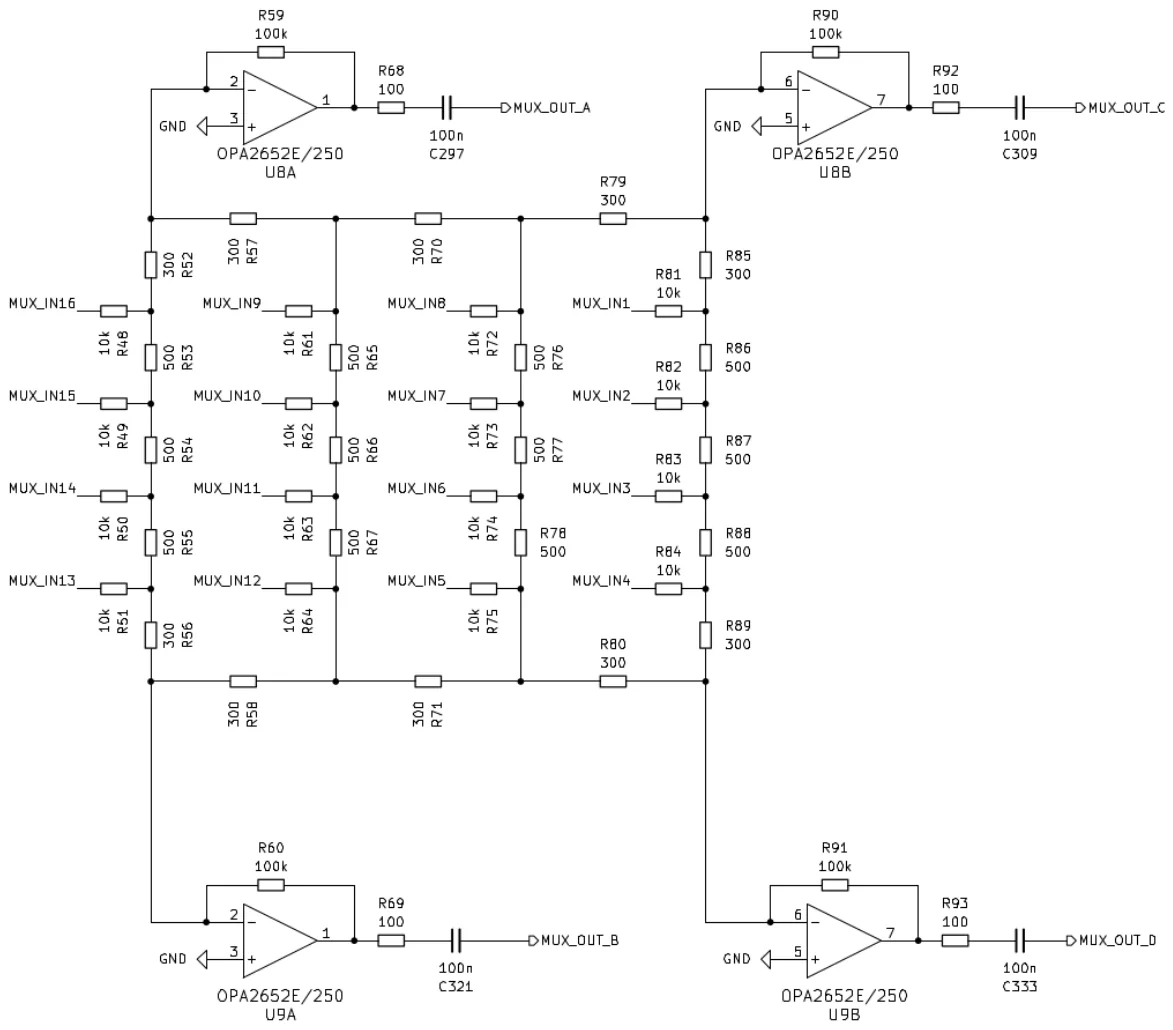
Resistive multiplexing network used to encode 16 SiPM input nodes into four output channels *A*, *B*, *C* and *D*. The relative charge collected at the four outputs is used for normalized charge division position reconstruction.

A resistively multiplexed SiPM array was studied as a channel-reduction strategy. The tested array used 16 Broadcom AFBR-S4N44P014M SiPMs, multiplexed into four output channels denoted *A*, *B*, *C* and *D*. The scheme follows a 16-to-4 charge division architecture in which the relative distribution of charge among the four outputs encodes the position of the activated SiPM node [13,14]. The network used resistors in the range from 300 Ω to 10 kΩ in a symmetric ladder configuration, as illustrated in Figure 3. The purpose of this layout is to provide a monotonic mapping between input position and output charge ratios while keeping the response of the different SiPM nodes as uniform as possible.

The reduction from 16 input nodes to four output channels corresponds to a factor-of-four reduction in the number of DAQ channels. In a full scintillating fibre detector, where large channel counts are expected, such a reduction simplifies cabling, front-end electronics, and data acquisition. The consequence of this simplification is that individual SiPM signals are no longer measured independently. The interaction position and signal amplitude must instead be inferred from the shared outputs. The performance of the method, therefore, depends on the stability of the charge sharing, the signal-to-noise ratio, the gain matching of the SiPMs and the calibration of the four output channels.

Although good timing performance is an important requirement for the intended FIMP detector, timing resolution was not investigated in this study. If the proposed multiplexing scheme is implemented in FIMP, the multiplexed outputs would primarily be used for SiPM identification and signal reconstruction, whereas event timing would be obtained through a separate parallel signal path. In this path, the SiPM signals would be combined using an inverting summing amplifier to provide a common timing signal independently of the multiplexing network. This separation is motivated by the signal division, additional noise and RC loading associated with the multiplexing network, which is expected to degrade the timing characteristics of signals propagated through the multiplexed path. A characterization of the dedicated timing channel therefore falls outside the scope of this work.

### 2.4. Pulsed Light Test Setup

The multiplexed SiPM readout was tested in a light-tight laboratory setup using localized pulsed light excitation. A light-emitting diode (LED) driven by a Rigol DG1062Z (RIGOL Technologies Co., Ltd., Suzhou, China) arbitrary waveform generator through a pulse-shaping circuit was coupled to an optical fibre and used to illuminate selected SiPM positions via a custom mechanical holder. This configuration provided good control over the input amplitude and allowed the response of individual SiPM nodes to be mapped systematically.

The SiPMs were biased at 36 V by an Aim-TTi PLH250-P (Thurlby Thandar Instruments Ltd., Huntingdon, UK) power supply and the rest of the analog circuits powered by a Rigol DP832A (RIGOL Technologies Co., Ltd., Suzhou, China) power supply. Output waveforms from the four multiplexed channels were sampled at 625 MS/s and 10-bit resolution using a PicoScope 6824E (Pico Technology Ltd., St Neots, UK) oscilloscope and processed offline. For each illumination configuration, approximately 1000 waveforms were recorded. The LED pulse width was 16 ns, and the LED circuit driving amplitude was varied over the range 5.80–20.00 V in steps of 50 mV between 5.80–10.00 V and steps of 250 mV between 10.25–20.00 V. These voltage settings define controlled pulsed light amplitudes and are not direct equivalents of deposited radiation energy. Calibration with scintillation signals from charged particles will therefore be required for the final detector response.

**Figure 4:**
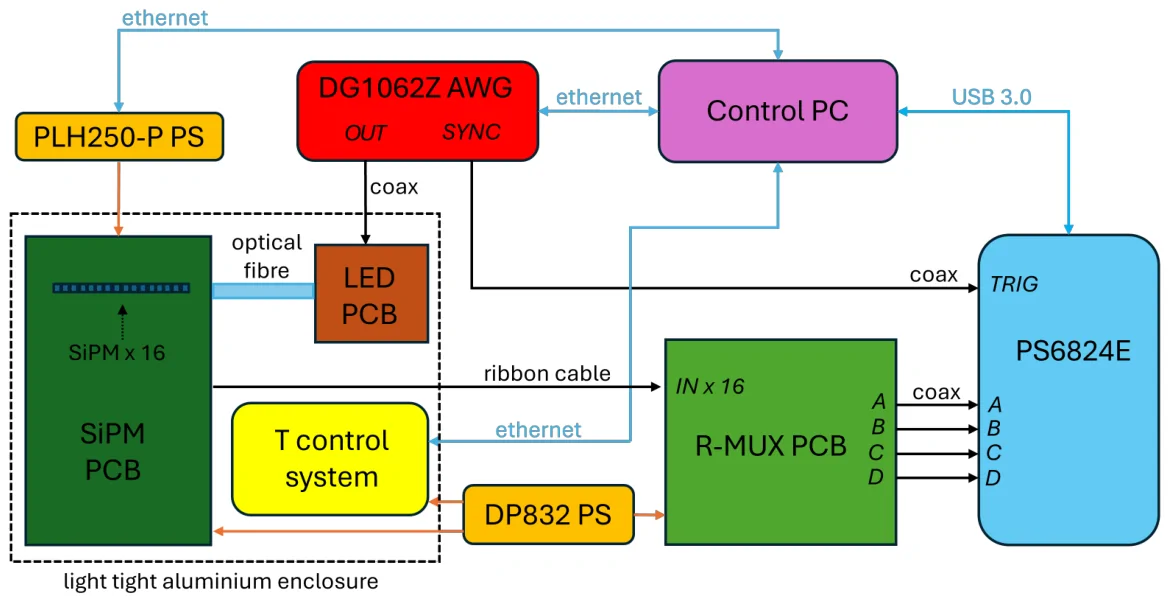
Pulsed light test setup scheme.

The overall test setup scheme is illustrated in Figure 4.

### 2.5. Signal Processing and Charge Division Reconstruction

For each event, the four output waveforms were processed using the same analysis sequence. A baseline value was determined from a pre-signal region and subtracted from each waveform. The signal charge equivalent in each output channel was then obtained by trapezoidal integration of the baseline-corrected waveform. The resulting integrated charge equivalents are denoted by *A*, *B*, *C* and *D*, and expressed in volt-nanoseconds.

The total signal was defined as

(6) Z=A+B+C+D.

The normalized charge division coordinates were calculated as

(7) $$X=\frac{\left(B+D)-(A+C\right)}{A+B+C+D},$$

and

(8) $$Y=\frac{\left(A+B)-(C+D\right)}{A+B+C+D}.$$

These coordinates reduce the first-order dependence on the total signal amplitude and encode the relative charge sharing between the four outputs. Events with small *Z* are more sensitive to baseline fluctuations and were therefore used to assess the low-amplitude reconstruction limit.

To quantify the size and separation of the reconstructed position distributions, the (X,Y) distribution obtained for each SiPM and input signal amplitude was characterized by a 99% probability content ellipse. The ellipse semi-major and semi-minor axes are denoted by *a* and *b*, respectively, and the ellipse area was defined as

(9) $$A_{xy}=\pi ab.$$

$A_{xy}$ was used as a measure of the spread of the reconstructed positions. For neighbouring SiPMs, $d_{\mathrm{NN}}$ denotes the distance between the ellipse centroids, while $g_{\mathrm{NN}}$ denotes the edge-to-edge gap between the corresponding ellipses. The normalized separation was defined as

(10) $$S=\frac{d_{\mathrm{NN}}}{\rho_{i}+\rho_{j}},$$

where $\rho_{i}$ and $\rho_{j}$ are the distances from the two ellipse centroids to their respective boundaries along the line joining the centroids. Thus, S>1 indicates separated ellipses, while S=1 corresponds to touching ellipse boundaries. Mean neighbour quantities were calculated over the 24 nearest-neighbour pairs of the 4×4 SiPM arrangement. Minimum values of $g_{\mathrm{NN}}$ and *S* were used to characterize the worst-separated neighbour pair at each input amplitude. For the ellipse area, $\bar{A_{xy}}$ denotes the mean over the 16 SiPMs and $A_{xy,\max}$ denotes the largest individual SiPM ellipse area at a given input amplitude.

The spread of the total signal proxy was characterized by its mean $Z_{\mathrm{avg}}$ and standard deviation $\sigma_{Z}$ for each SiPM and input amplitude. The relative *Z* spread for an individual SiPM was defined as

(11) $$r_{Z}=\frac{\sigma_{Z}}{Z_{\mathrm{avg}}},$$

and the value reported in Table 2, $\bar{\sigma_{Z}/Z_{\mathrm{avg}}}$, is the mean of this quantity over the 16 SiPMs.

${\mathrm{SNR}}_{Z,\mathrm{int}}$ denotes the integral-based signal-to-noise ratio of the total signal proxy *Z*. For each SiPM and input amplitude, a mean SNR was obtained from the valid events. The mean SNR reported in Table 2, $\bar{{\mathrm{SNR}}_{Z,\mathrm{int}}}$, is the mean of these values over the 16 SiPMs, while ${\mathrm{SNR}}_{Z,\mathrm{int},\min}$ is the lowest individual SiPM mean SNR at that input amplitude.

The minimum separation amplitude, $V_{\mathrm{sep}}$, was defined as the lowest tested LED driving amplitude for which none of the 16 reconstructed 99% ellipses overlapped. To characterize the stability of the reconstructed positions at larger signals, the ellipse obtained at $V_{\mathrm{sep}}$ was subsequently used as a reference ellipse for each SiPM. The escape amplitude, $V_{\mathrm{esc}}$, was defined as the first higher tested amplitude at which the centroid of a given SiPM moved outside its reference ellipse. The corresponding amplitude margin was defined as

(12) $$\Delta V_{\mathrm{esc}}=V_{\mathrm{esc}}-V_{\mathrm{sep}}.$$

## 3. Results

**Figure 5:**
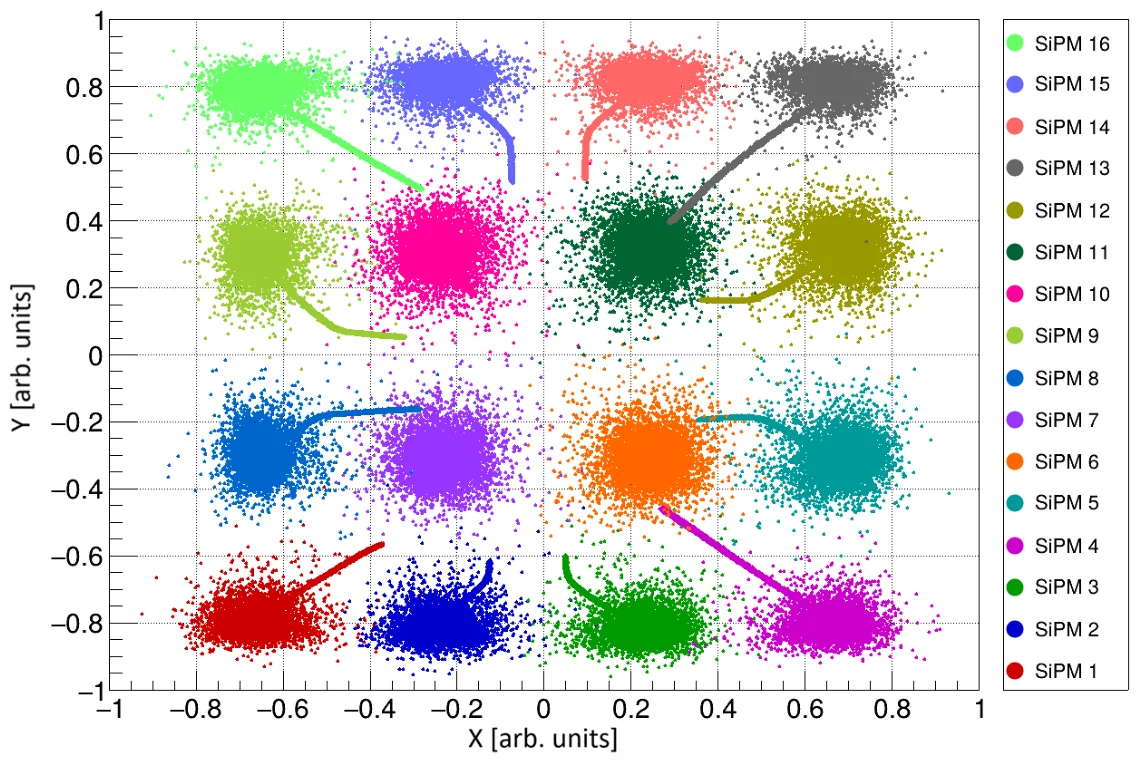
Reconstructed position map for the 16 SiPM input positions using normalized charge division coordinates. The colour coding corresponds to the illuminated SiPM position. Distinct clusters indicate that the 16 input nodes remain distinguishable after reduction to four output channels.

The reconstructed (X,Y) distributions for the 16 SiPM illumination positions are shown in Figure 5. Distinct clusters are visible for the individual input nodes, indicating that the four-output resistive network retains spatial information under suitable operating conditions. The cluster topology reflects the mapping of the SiPM nodes through the resistive ladder and shows that the normalized charge asymmetry method provides a usable position estimator for the 16-to-4 architecture.

At intermediate signal amplitudes, the reconstructed clusters were compact and separated. This was the most favourable region observed in the present measurements because the signal-to-noise ratio was sufficiently high while the SiPM response and resistive network remained approximately linear. In this range, the observed clustering supports considering multiplexed SiPM readout as a compact option for position-sensitive scintillating fibre detectors.

**Figure 6:**
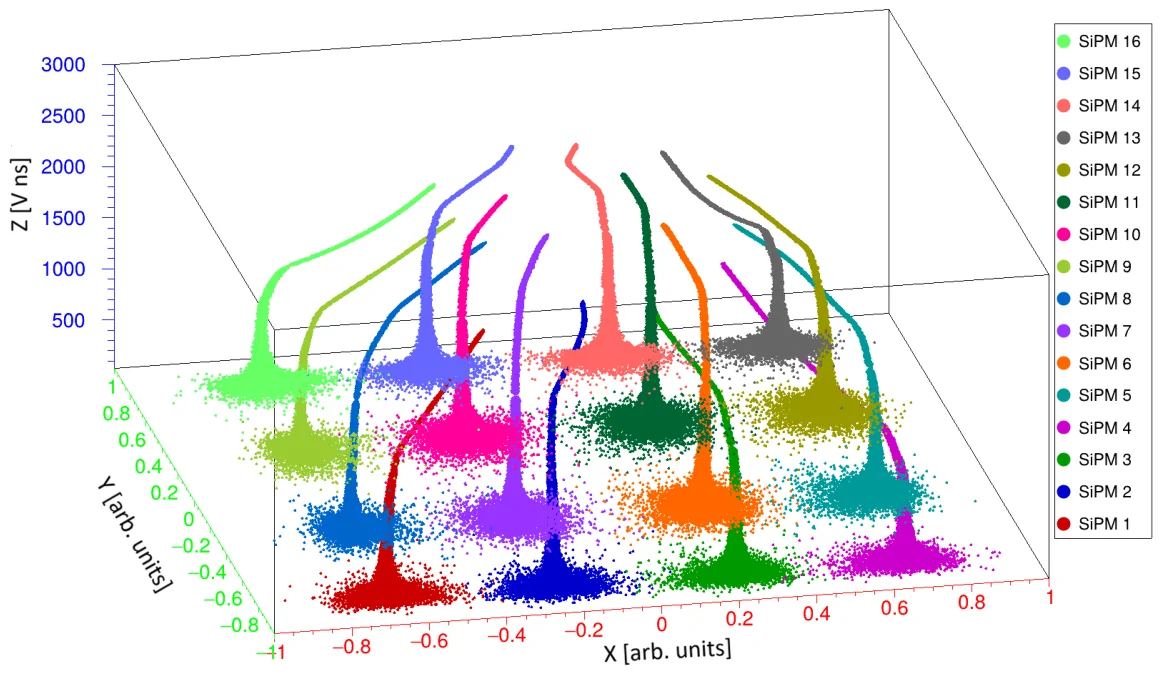
Three-dimensional representation of the reconstructed response, showing the normalized position coordinates together with the total signal proxy *Z* for the practical signal amplitude range. This view is useful for assessing whether position and amplitude information remain correlated or separable after resistive multiplexing.

A three-dimensional representation using *X*, *Y* and the total signal *Z* is shown in Figure 6. This representation displays position and amplitude simultaneously. In a calibrated radiation detector, such information may help separate event classes, for example, beta-like low-amplitude events, alpha-like events and larger ion implantation signals. One has to stress, though, that this interpretation requires calibration with radioactive sources or beam data and is not inferred from LED data alone.

**Figure 7:**
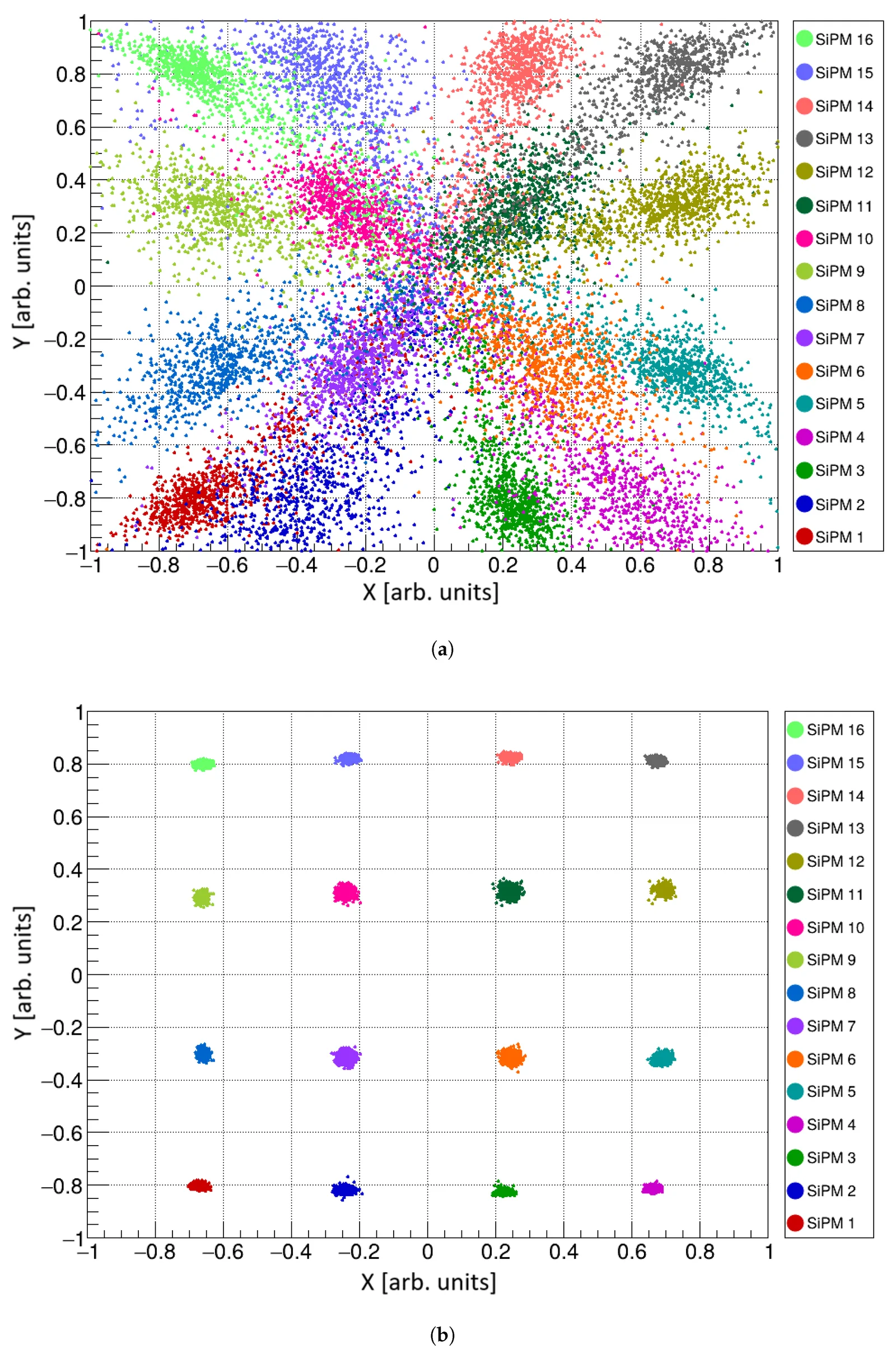
Effect of front-end noise on reconstructed position. (**a**) High-noise configuration with elongated reconstructed clusters and large coordinate excursions. (**b**) Lower-noise configuration after improved grounding and readout implementation.

Noise was one of the main limitations at low signal amplitudes. In the initial high-noise configuration, the reconstructed clusters showed elongation and broad tails, as illustrated in Figure 7a. Such behaviour is expected when baseline fluctuations and noise pickup become comparable to the integrated signal. Because the position coordinates in Equations (7) and (8) are normalized by *Z*, even moderate fluctuations in the individual channels can produce large apparent coordinate excursions when *Z* is small.

The elevated noise in the initial configuration was attributed primarily to switching power supply interference elsewhere in the experimental setup, which produced substantial pulsed noise pickup that could not be adequately removed by offline filtering. Replacing this supply and improving the readout implementation reduced the mean RMS baseline noise from $\sigma_{\mathrm{base},\mathrm{RMS}}=23.1\;\mathrm{mV}$ to 7.1mV and increased ${\mathrm{SNR}}_{Z,\mathrm{int}}$ from 7.4 to 395.6 at the same LED driving amplitude of 8.4 V.

The reconstructed distribution for a configuration with lower noise pickup at equivalent incident light intensities is shown in Figure 7b. It retains a more compact cluster topology, indicating that stable analog signal handling is essential for multiplexed charge division readout.

The low-amplitude limit is particularly relevant for beta-decay detection. If the signal produced by a beta particle in a fibre is close to the detection threshold, the reconstructed total signal can become comparable to the baseline uncertainty. In this regime, cluster overlap and unstable normalized coordinates are expected. A robust operating threshold must therefore be defined not only from the detectability of a signal in one channel but from the ability to reconstruct a stable position after multiplexing.

**Figure 8:**
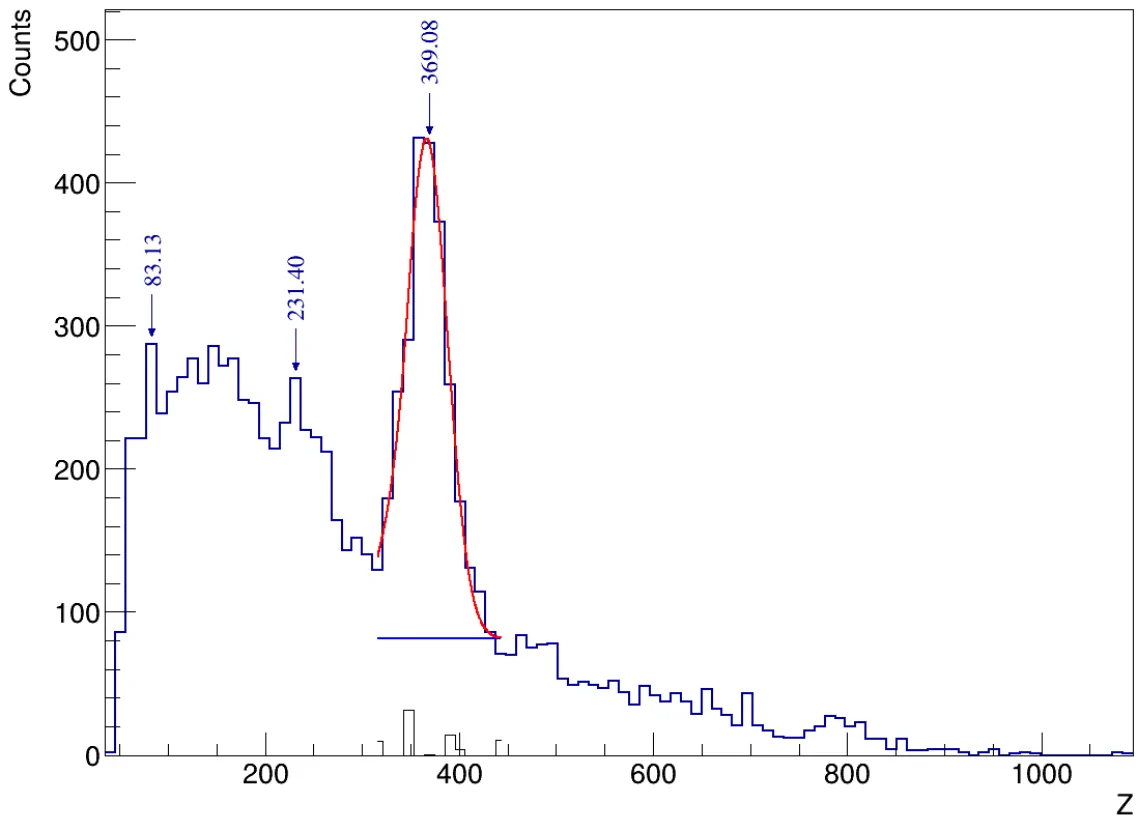
Qualitative ^22^Na source measurement obtained with a $2\times 2\times 20\;{\mathrm{mm}}^{3}$ LYSO crystal coupled to SiPM 16. The prominent feature at Z≈369 was tentatively assigned to the 511-keV annihilation photopeak and used only as an approximate signal-scale reference when selecting the SiPM operating conditions. The horizontal axis is not energy-calibrated. The red line represents a tentative Gaussian peak fit.

Before the systematic LED measurements, a brief radioactive source test was performed solely to select a practical SiPM bias voltage. A ^22^Na source was placed next to a $2\times 2\times 20\;{\mathrm{mm}}^{3}$ LYSO crystal optically coupled to SiPM 16, and several candidate bias settings were assessed qualitatively in terms of signal visibility, noise, and available dynamic range. Based on this preliminary check, a bias voltage of 36 V was selected for the subsequent measurements. At this operating point, the measured total-signal distribution contained a prominent feature at Z≈369, shown in Figure 8, tentatively assigned to the 511keV annihilation photopeak based on the evaluated ^22^Na decay data [29]. This measurement was used only to establish an approximate signal scale and choose a suitable SiPM operating point. The measurement was not used to establish an energy calibration, linearity, energy resolution, or detection efficiency. Accordingly, Z≈369 should not be interpreted as defining a calibrated conversion between *Z* and deposited energy.

**Figure 9:**
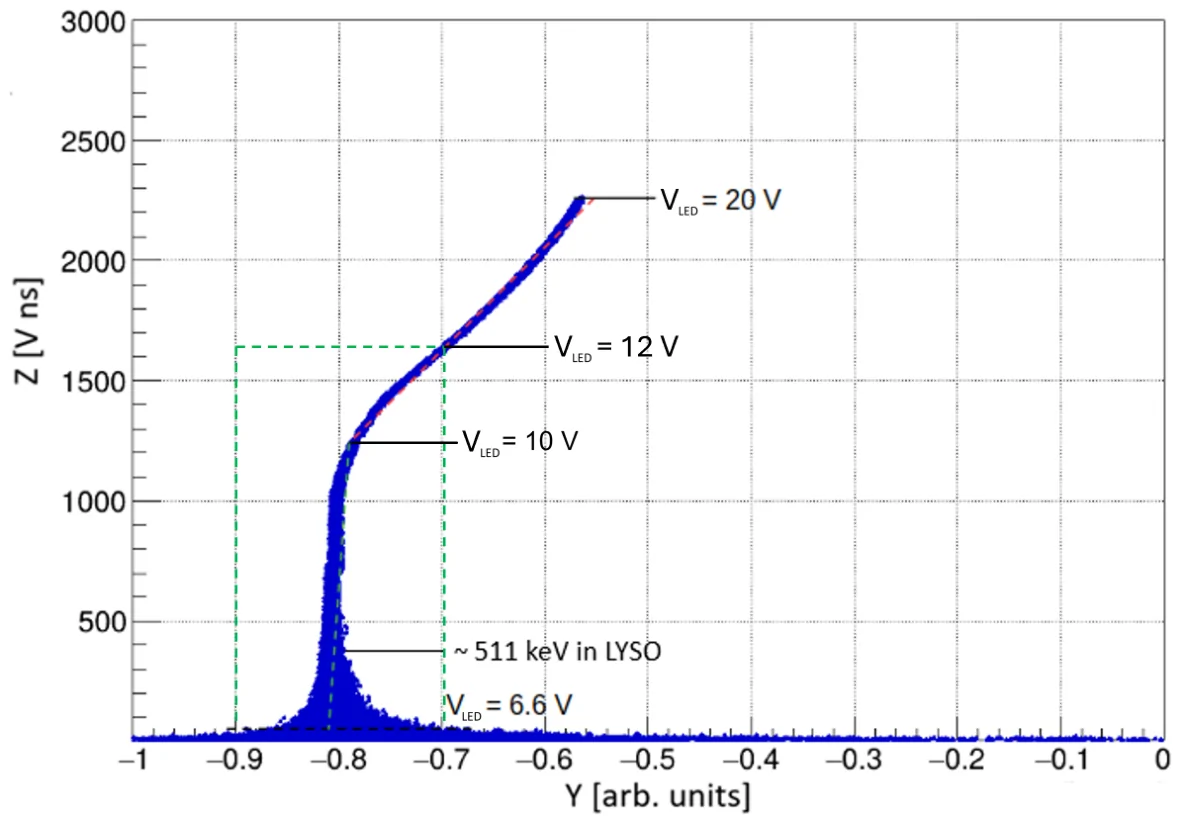
Dependence of the reconstructed response on LED driving amplitude for SiPM 1. The tested amplitudes include the low-signal regime, an intermediate regime with stable reconstruction and a high-amplitude regime in which non-linear effects become visible. The plotted peak-to-peak voltage values correspond to LED drive settings, not directly to deposited radiation energy. The green dashed line represents the YZ projection region within which the reconstructed position remains inside the ellipse defined at $V_{\mathrm{LED}}=V_{\mathrm{sep}}=6.70$ V.

The response of the readout was then studied as a function of LED driving amplitude. Figure 9 shows the reconstructed response for several LED settings. At the lowest amplitudes, the reconstructed distribution is dominated by noise and threshold effects. At intermediate amplitudes, compact and stable cluster regions were obtained. At the highest amplitudes, the response is distorted by non-linear effects. These observations identify an operating window defined by noise at low signals and by saturation or non-linear signal sharing at high signals.

**Figure 10:**
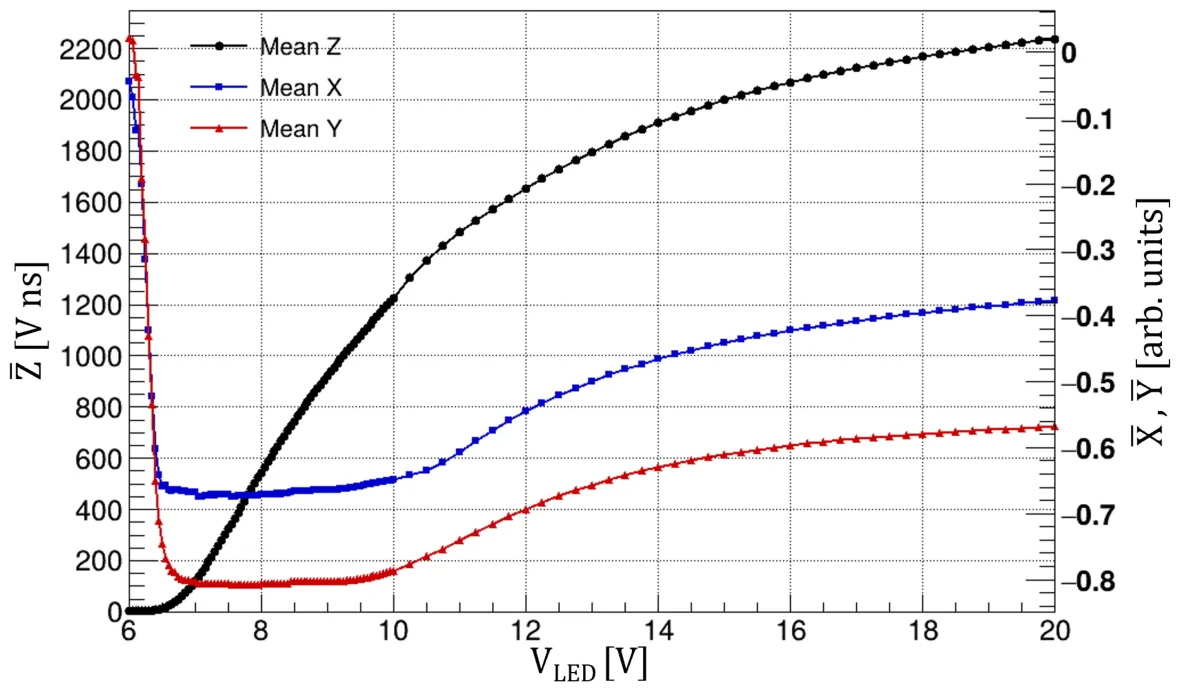
Dependence of the mean reconstructed *Z*, *X*, and *Y* on LED driving amplitude for SiPM 1.

**Figure 11:**
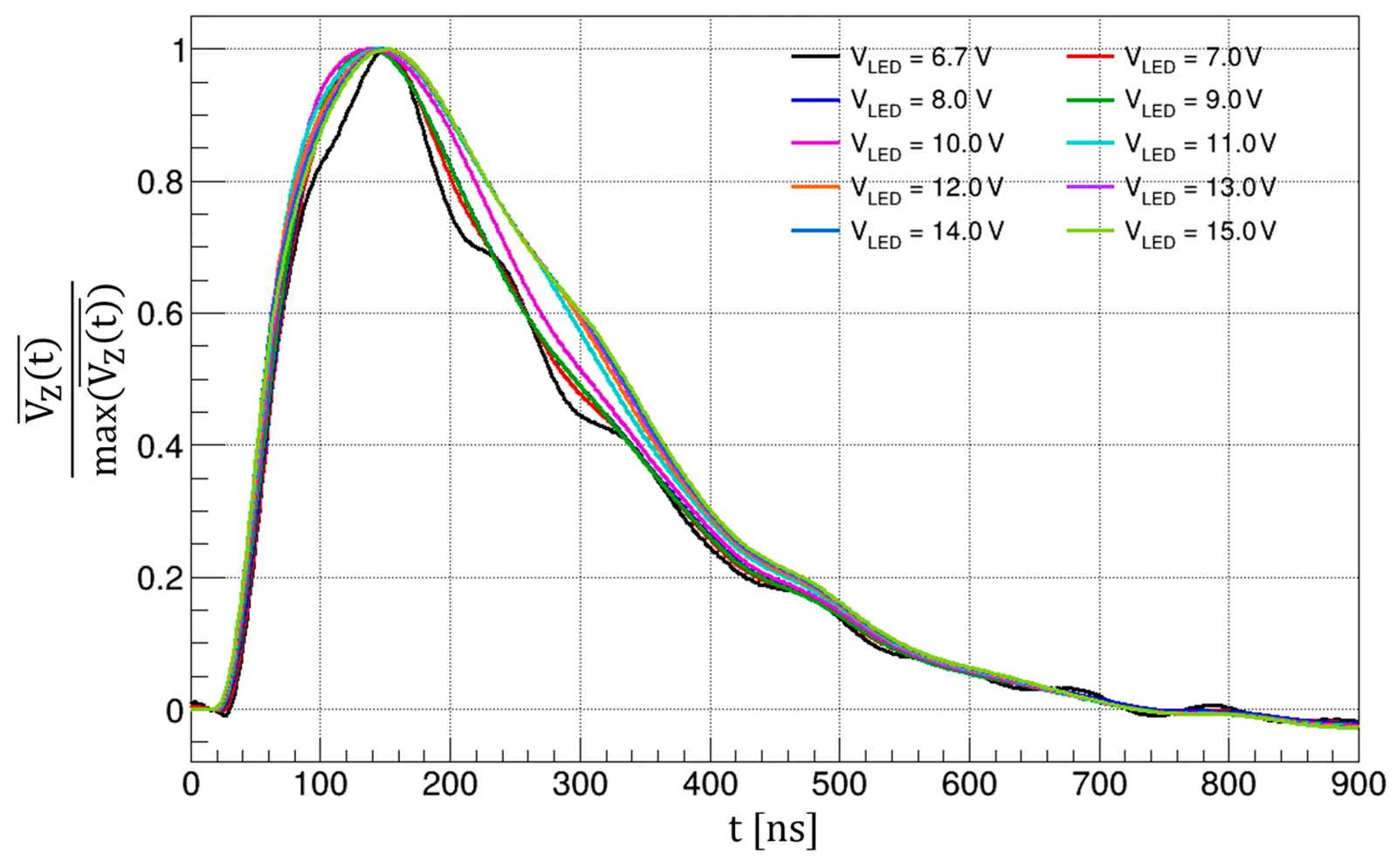
Peak-normalized mean total signal proxy $V_{Z}$ waveforms for SiPM 1 over a range of $V_{\mathrm{LED}}$ values.

**Figure 12:**
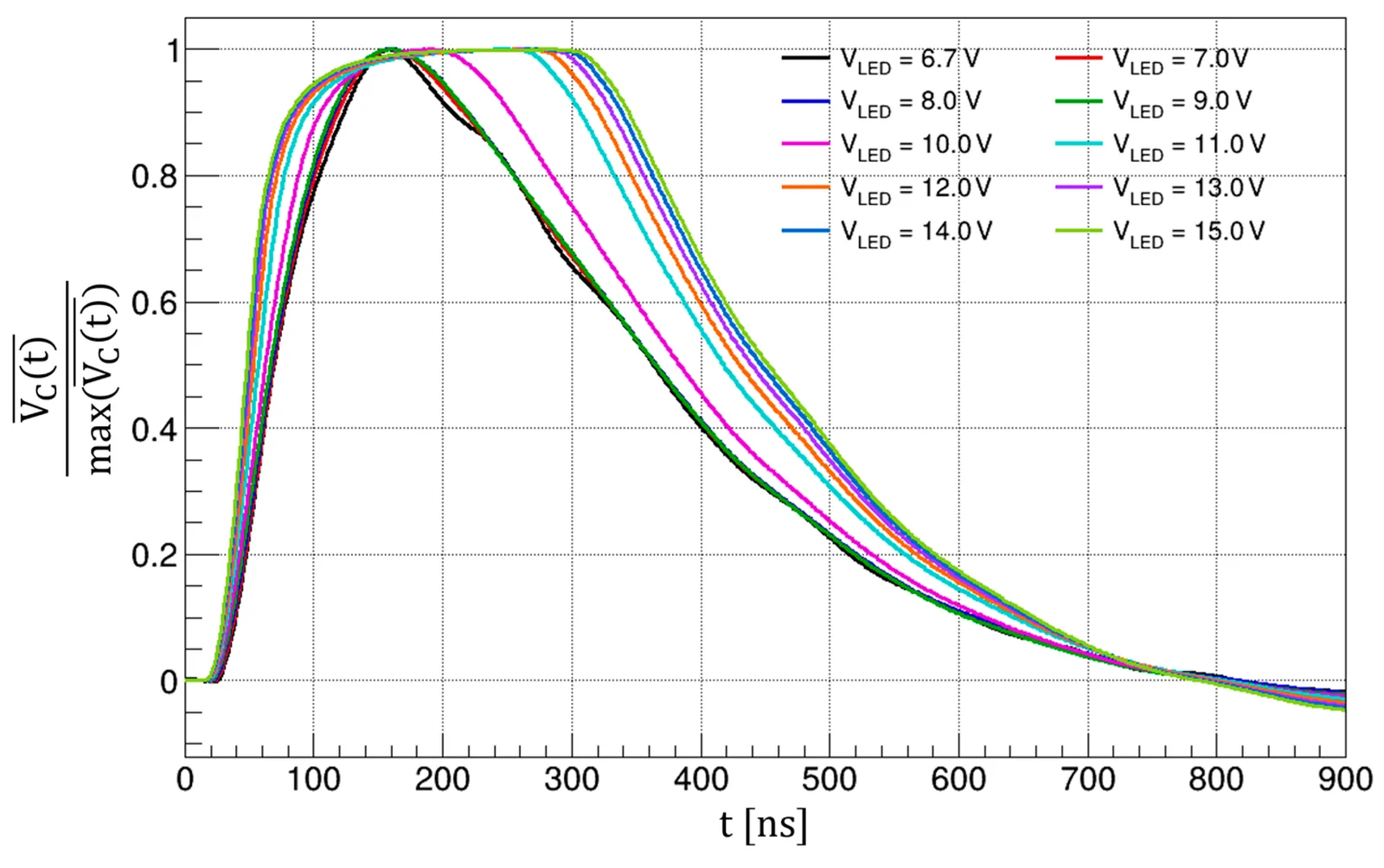
Peak-normalized mean $V_{C}$ waveforms for SiPM 1 over a range of $V_{\mathrm{LED}}$ values.

The total signal *Z* increased with increasing input amplitude, but significant deviations in the XY centroid position were observed at higher amplitudes, beginning at approximately $V_{\mathrm{LED}}=10.0$ V. Such deviations could originate from changes in LED luminosity, SiPM saturation due to finite microcell recovery, amplifier saturation, or non-linear response of the multiplexing network. To determine the cause of the XY drift, changes in *Z*, *X*, and *Y* were investigated separately. Figure 10 shows the dependence of the mean reconstructed *Z*, *X*, and *Y* on $V_{\mathrm{LED}}$ in the range between 6.00–20.00 V. While the Z response is nonlinear with respect to $V_{\mathrm{LED}}$, its change is gradual and does not account for the fairly significant tilt in the YZ projection in Figure 9. *X* and *Y* responses, however, show distinct drifts from values established at the reference $V_{\mathrm{LED}}$ level of 6.7 V, starting at $V_{\mathrm{LED}}$ = 10.0 V. This indicates a change in the charge division ratios of the multiplexing matrix. This can also be observed in the averaged total signal proxy $V_{Z}\left(t\right)=V_{A}\left(t\right)+V_{B}\left(t\right)+V_{C}\left(t\right)+V_{D}\left(t\right)$ waveforms and averaged $V_{C}\left(t\right)$ waveforms for a range of $V_{\mathrm{LED}}$ values, shown in Figure 11 and Figure 12, respectively. In both cases, the waveforms were normalized to their amplitude, enabling the comparison of signal shapes corresponding to different input amplitudes. $V_{C}$ was chosen because SiPM 1 signals enter the multiplexing matrix closest to output node *C* (referring to Figure 3), meaning that it will receive the largest share of the input charge. The shape of $V_{Z}$ pulses again varies gradually with increasing $V_{\mathrm{LED}}$, suggesting no significant sharp change in distortion occurs in the SiPM signal entering the resistive multiplexing matrix. $V_{C}$ pulses, however, show a distinct rapid change in signal shape towards a flat-topped pulse occurring at approximately $V_{\mathrm{LED}}$ = 10.0 V. This points towards saturation of the output amplifier in the *C* output node of the multiplexing matrix. The conclusion is consistent with changes in cluster reconstructions seen in Figure 5 and Figure 6, showing that the onset and degree of distortion depend on the location of the SiPM within the multiplexing matrix.

For radiation detection, this amplitude dependence has direct consequences. The low-amplitude boundary is expected to contribute to beta sensitivity and to the probability of losing decay events close to threshold. The operating point is therefore chosen by balancing SiPM overvoltage, light collection, front-end gain and the required dynamic range.

### Quantitative Reconstruction Performance

Complete separation of the 16 reconstructed position regions was first obtained at

(13) $$V_{\mathrm{sep}}=6.70\;V.$$

At the preceding tested amplitude of 6.65 V, eight overlapping ellipse pairs were still present. The reconstruction characteristics at $V_{\mathrm{sep}}$ and at the maximum investigated LED driving amplitude of 20.00 V are summarized in Table 2. In addition to mean quantities, maximum ellipse area and minimum neighbour-separation values are reported to characterize the worst-case response of the array.

Because *X* and *Y* are normalized coordinates, the corresponding distances are dimensionless and $A_{xy}$ is expressed in squared normalized-coordinate units. At the minimum complete-separation amplitude of 6.70 V, the mean reconstruction-ellipse area was 0.0877, while the largest individual ellipse had an area of 0.1695. The mean nearest-neighbour edge gap was 0.1420 and the minimum gap was 0.0656. The corresponding mean normalized separation was 1.4169, with a minimum value of 1.1811. The fact that $S_{\min}>1$ is consistent with all nearest-neighbour reconstruction ellipses being separated at this amplitude. At the maximum tested amplitude of 20.00 V, the reconstruction ellipses were substantially smaller, with a mean area of 0.0001 and a maximum area of 0.0002. Although the mean nearest-neighbour centroid distance decreased from 0.4909 to 0.2932, the reduction in ellipse size increased the mean edge gap to 0.2804 and the minimum edge gap to 0.1506. The mean normalized separation increased to 27.3262, while even the worst-separated neighbouring pair had $S_{\min}=10.5961$. The reconstruction regions therefore remained clearly separated throughout the measured range above $V_{\mathrm{sep}}$. The integral-based total-signal SNR also increased strongly with input amplitude. At 6.70 V, the mean SNR across the 16 SiPMs was 31.1 and the lowest individual SiPM mean SNR was 24.0. At 20.00 V, these values increased to 1128.9 and 930.8, respectively. At the same time, the mean relative spread $\bar{\sigma_{Z}/Z_{\mathrm{avg}}}$ decreased from 10.04% to 0.35%, showing that the relative event-to-event variation of the total signal became much smaller at high signal amplitudes. The position stability at higher amplitudes was evaluated relative to the 99% reconstruction ellipse obtained at $V_{\mathrm{sep}}=6.70$ V. The first amplitude at which the centroid of each SiPM moved outside its corresponding reference ellipse is listed in Table 3.

The first reference ellipse crossing was observed for SiPM 13 at 10.50 V, corresponding to an amplitude margin of 3.80 V above the minimum complete-separation point. SiPMs 6, 7, 10, and 11 remained inside their respective reference ellipses throughout the investigated range and are therefore reported as $V_{\mathrm{esc}}>20.00$ V. The escape amplitude does not represent a loss of SiPM separability. Instead, it indicates that the mean reconstructed position has shifted outside the 99% region determined at 6.70 V. Despite these centroid shifts, the reconstruction regions remained mutually separated up to the maximum investigated amplitude.

**Figure 13:**
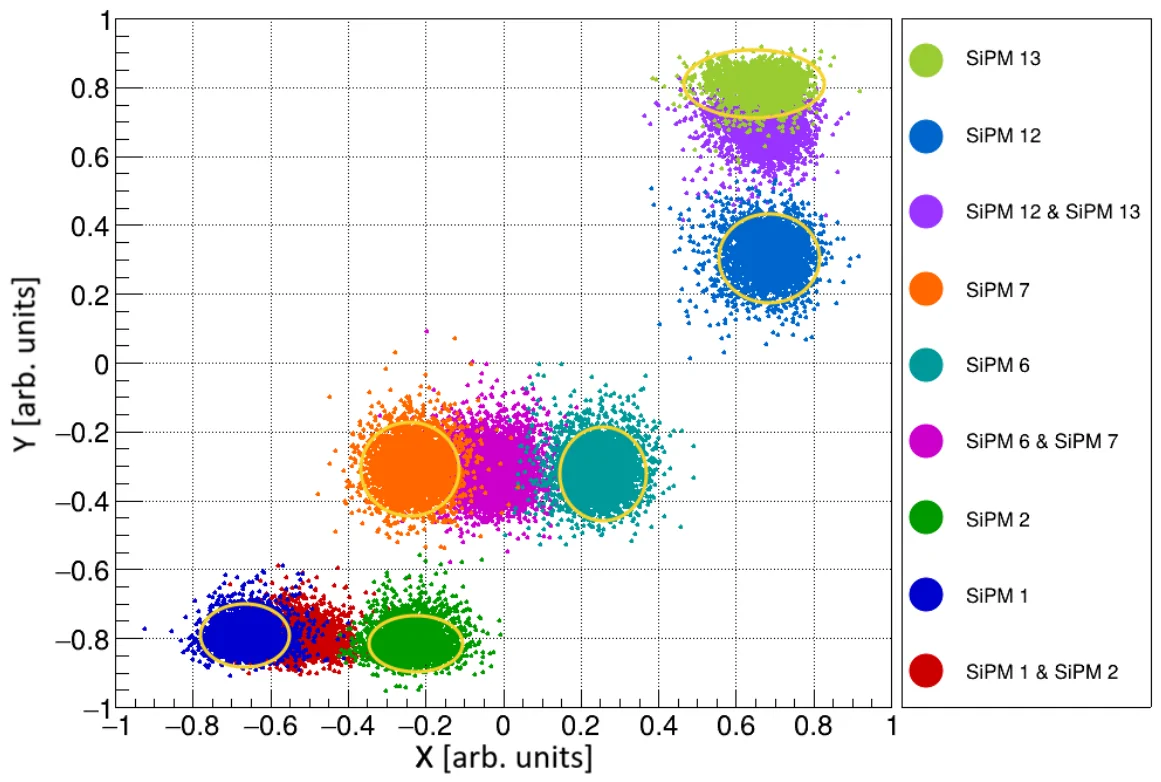
Reconstructed position map of events with simultaneous illuminations of pairs of SiPMs. Clusters corresponding to proper SiPM node reconstructions are circled in yellow for reference.

Radiation events in a scintillating fibre detector may produce light in several neighbouring fibres or be coupled to several SiPM inputs simultaneously. This is especially relevant for implanted ions, which produce extended tracks, and for particles crossing more than one fibre. Additional laboratory tests were therefore performed with simultaneous excitation of several SiPM inputs. The corresponding position maps showed spatially extended or shifted distributions rather than single-node clusters, as shown in Figure 13.

This behaviour is consistent with the multiplexing principle. The reconstructed coordinates represent the charge-weighted response of the activated nodes. For single-fibre or single-node excitation, the result is a cluster associated with one input position. For multi-node excitation, the reconstructed position moves toward the charge-weighted centroid of the active nodes. In a complete detector, this effect could be useful for reconstructing extended ion tracks, but it also means that event topology reconstruction will require calibration or simulation of the fibre response, optical cross-talk and charge sharing.

**Figure 14:**
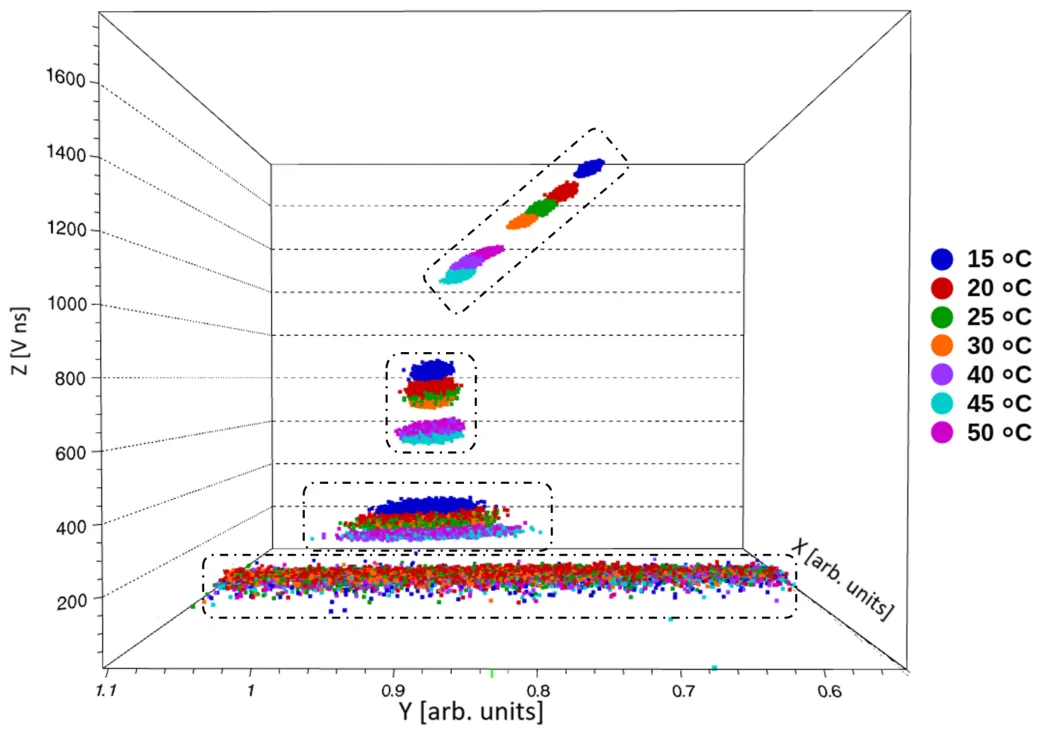
Preliminary temperature-dependence test of the reconstructed response. Position reconstruction clusters corresponding to equal $V_{\mathrm{LED}}$ values are grouped in dashed boxes. Temperature-dependent changes in SiPM gain and noise can affect the charge division coordinates and therefore require stabilization or calibration.

The SiPM gain, dark count rate and breakdown voltage are temperature dependent [21,22]. At fixed bias voltage, temperature variations change the effective overvoltage and therefore the gain, while the dark count rate modifies the noise contribution. These changes can affect the reconstructed position if the four output channels are not affected identically. A temperature-dependence test was performed over a range of set temperatures for a single SiPM. The SiPM circuit board and LED pulser were placed in a thermally insulated box with an active temperature control system based on Peltier elements and PT100 temperature sensors. They were allowed to thermalise for 30 min before performing measurements at four different LED pulse amplitudes. The results are shown in Figure 14, with the data points corresponding to equal pulse amplitudes at different temperatures circled together. The test indicates that position reconstruction at different temperatures is possible by taking changes in the total signal proxy Z into account, as the effect of temperature variations is mostly equivalent to changes in input signal amplitudes, due to the positive temperature coefficient of the SiPM breakdown voltage [21], changes to noise contributions notwithstanding. Proper energy estimation for event reconstruction in a practical detector application would, however, require calibration or temperature stabilization.

The temporal stability of the multiplexing scheme and position reconstruction was not systematically investigated; however, no noteworthy changes were observed during the five-day measurement period.

## 4. Discussion

Two physical limits define the usable operating range of the resistively multiplexed SiPM readout. At low amplitudes, the limiting factor is the signal-to-noise ratio. Baseline fluctuations, grounding pickup, dark count contributions and integration uncertainty broaden the reconstructed clusters and can make the normalized coordinates unstable. This limit is particularly important for beta detection, because beta particles can deposit relatively small amounts of energy in individual fibres.

At high amplitudes, the limiting factor is non-linearity. The SiPM response is only quasi-linear while the number of fired microcells remains small compared to the number of available cells. Once the microcell occupancy increases, additional photons no longer produce proportional charge. Larger currents through the resistive network can also introduce voltage drops or unequal response among the four outputs. Amplifiers used in multiplexing scheme signal chain may enter into saturation depending on the exact amplifier model used, and the applied supply voltages. All these effects modify the relative charge sharing and can shift the reconstructed coordinates. A simple total-charge threshold is therefore not sufficient. The reconstruction quality has to be evaluated as a function of both signal amplitude and position.

The quantitative reconstruction analysis confirms that the useful response range cannot be characterized by signal amplitude alone. Complete separation of all 16 reconstructed regions was first obtained at an LED driving amplitude of 6.70 V. Above this point, the reconstruction ellipses became progressively smaller and remained mutually separated up to the maximum investigated amplitude of 20.0 V. However, the centroids of several SiPM reconstruction regions shifted with increasing amplitude. The lower end of the useful range is therefore primarily associated with noise-induced cluster broadening and overlap, whereas the higher-amplitude behaviour is characterized mainly by systematic changes in the reconstructed position rather than by renewed overlap of the reconstruction regions. The operating range can therefore be characterized using the measured cluster separation and amplitude-dependent centroid stability. In a practical detector, these quantities can be evaluated as part of the same calibration procedure used to establish the charge division map.

The channel-count reduction studied here is directly relevant for scalable scintillating fibre radiation sensors. Reducing 16 SiPM input nodes to four output channels simplifies the digitization chain and reduces the number of cables and front-end inputs. This is advantageous for compact detector assemblies embedded inside γ-ray and neutron detector arrays, where space and material budget are constrained.

The consequence of this simplification is a stronger dependence on calibration and reconstruction. The detector response must be mapped, and gain variations among SiPMs, optical-coupling non-uniformities and temperature effects must be corrected. In a full detector, the charge division map should therefore be treated as part of the sensor calibration, not only as an electronics feature.

For FIMP, the relevant application is implantation–decay correlation. The detector must detect high-amplitude ion implantations and subsequent lower-amplitude decay particles in the same active volume. A multiplexed readout is useful if it retains sufficient spatial information for both event classes. The LED measurements provide a controlled characterization of the readout network.

The use of orthogonal fibre layers also means that a complete detector event will involve correlations between two or more readout planes. The present 16-to-4 characterization addresses one multiplexed sensor plane or module. In a full detector, reconstruction should combine charge division coordinates from multiple planes with timing and amplitude information. This multi-plane information may improve background rejection, distinguish true decay events from cross-talk-induced hits and reduce false implantation–decay correlations.

The present LED measurements should therefore be understood as a sensor-level characterization of the multiplexed readout rather than as a full detector response measurement. Controlled LED excitation does not reproduce the complete light-production, transport, and photon-counting statistics of scintillation events. In particular, the small relative spread of the total signal (*Z*) observed at high LED amplitudes should not be interpreted as representative of the resolution expected for low-light scintillating fibre signals, for which fluctuations in the number of detected photons will introduce additional broadening. The 16 ns LED drive pulse used in the present measurements also differs from the few-nanosecond emission times characteristic of plastic scintillating fibres [9,28]. Because finite SiPM microcell occupancy depends on the temporal distribution of the incident photons relative to the microcell recovery time, the longer LED excitation may shift the apparent onset of non-linearity to higher light levels than would occur for a faster scintillation pulse. Furthermore, realistic detector events may distribute light unequally among several fibres and SiPMs, whereas the present multi-node measurements were limited to simultaneously illuminated pairs. Fibre attenuation and longitudinal response variations, as well as particle-dependent scintillation quenching, were not included in the present study. These effects will require calibration and validation using complete scintillating fibre assemblies together with radioactive source or beam measurements.

## 5. Conclusions

A resistively multiplexed SiPM readout for position-sensitive scintillating fibre radiation detectors was characterized using controlled pulsed light excitation. The architecture combines 16 SiPM input nodes into four output channels, reducing the number of digitizer channels by a factor of four. Position reconstruction was performed using normalized charge division coordinates derived from the four integrated output signals. The reconstructed populations were characterized using 99% probability content ellipses and nearest-neighbour separation metrics.

The reconstructed charge asymmetry plane showed distinguishable clusters for the individual SiPM positions in an intermediate signal amplitude range. At low amplitudes, the reconstruction was limited by baseline noise and the reduced signal-to-noise ratio. Complete separation of the 16 reconstructed SiPM regions was achieved from an LED driving amplitude of 6.70 V, while the reconstruction regions remained mutually separated up to the maximum investigated amplitude of 20.0 V. At high amplitudes, saturation-like response and possible network-induced changes in charge sharing distorted the reconstructed coordinates with the first reference ellipse crossing occurring at 10.50 V. The useful operating range is therefore bounded by the noise-dominated regime at small signals and the non-linear regime at large signals.

The results support resistive multiplexing as a candidate compact readout strategy for SiPM-based scintillating fibre radiation detectors. For the FIMP detector concept, the method offers a possible route to reduced front-end complexity while retaining the position information needed for implantation–decay correlation. Future work will focus on temperature and gain corrections, calibration of the LED response with radioactive source or beam data, and evaluation of the multiplexing method with realistic scintillating fibre detector event topologies.

## Figures and Tables

**Table 2:** Summary of the multiplexing network reconstruction performance at the minimum complete-separation amplitude and at the maximum investigated input amplitude.

Quantity	$V_{\mathrm{in}}=6.70$ V	$V_{\mathrm{in}}=20.00$ V
Mean ellipse area, $\bar{A_{xy}}$	0.0877	0.0001
Maximum ellipse area, $A_{xy,\max}$	0.1695	0.0002
Mean NN centroid distance, $\bar{d_{\mathrm{NN}}}$	0.4909	0.2932
Mean NN edge gap, $\bar{g_{\mathrm{NN}}}$	0.1420	0.2804
Minimum NN edge gap, $g_{\mathrm{NN},\min}$	0.0656	0.1506
Mean normalized separation, $\bar{S}$	1.4169	27.3262
Minimum normalized separation, $S_{\min}$	1.1811	10.5961
Mean integral *Z* SNR, $\bar{{\mathrm{SNR}}_{Z,\mathrm{int}}}$	31.1	1128.9
Minimum integral *Z* SNR, ${\mathrm{SNR}}_{Z,\mathrm{int},\min}$	24.0	930.8
Mean relative *Z* spread, $\bar{\sigma_{Z}/Z_{\mathrm{avg}}}$	10.04%	0.35%

**Table 3:** Input signal amplitude at which the reconstructed centroid of each SiPM first leaves its 6.70 V reference ellipse.

SiPM	$V_{\mathrm{esc}}$ [V]	$\Delta V_{\mathrm{esc}}$ [V]
1	12.00	5.30
2	16.75	10.05
3	14.50	7.80
4	10.75	4.05
5	14.25	7.55
6	>20.0	>13.30
7	>20.0	>13.30
8	13.25	6.55
9	12.50	5.80
10	>20.0	>13.30
11	>20.0	>13.30
12	13.50	6.80
13	10.50	3.80
14	13.25	6.55
15	13.00	6.30
16	10.75	4.05

## Data Availability

The data presented in this study are available from the corresponding author upon reasonable request.
